# Cardiac and Vascular Remodeling After 6 Months of Therapy With Sacubitril/Valsartan: Mechanistic Insights From Advanced Echocardiographic Analysis

**DOI:** 10.3389/fcvm.2022.883769

**Published:** 2022-05-18

**Authors:** Sara Monosilio, Domenico Filomena, Federico Luongo, Michele Sannino, Sara Cimino, Matteo Neccia, Marco Valerio Mariani, Lucia Ilaria Birtolo, Giulia Benedetti, Giovanni Tonti, Gianni Pedrizzetti, Carmine Dario Vizza, Viviana Maestrini, Luciano Agati

**Affiliations:** ^1^Department of Clinical, Internal, Anesthesiological and Cardiovascular Sciences, “Sapienza” University of Rome, Policlinico Umberto I, Rome, Italy; ^2^Cardiology Division, ‘G. D'Annunzio’ University, Chieti, Italy; ^3^Department of Engineering and Architecture, University of Trieste, Trieste, Italy

**Keywords:** sacubitril/valsartan, echocardiography, speckle-tracking, hemodynamic forces, pressure-volume loop

## Abstract

**Background:**

Effects of Sacubitril/Valsartan (S/V) on left ventricular (LV) mechanics and ventricular-arterial coupling in patients with heart failure with reduced ejection fraction (HFrEF) are not completely understood. The aim of this study was to evaluate both cardiac and vascular remodeling in a group of HFrEF patients undergoing S/V therapy.

**Methods:**

Fifty HFrEF patients eligible to start a therapy with S/V were enrolled. Echocardiographic evaluation was performed at baseline and after 6 months of follow-up (FU). Beside standard evaluation, including global longitudinal strain (GLS), estimated hemodynamic forces (HDFs) and non-invasive pressure-volume curves (PV loop) were assessed using dedicated softwares. HDFs were evaluated over the entire cardiac cycle, in systole and diastole, both in apex to base (A-B) and latero-septal (L-S) directions. The distribution of LV HDFs was evaluated by L-S over A-B HDFs ratio (L-S/A-B HDFs ratio). Parameters derived from estimated PV loop curves were left ventricular end-systolic elastance (E_es_), arterial elastance (E_a_), and ventricular-arterial coupling (VAC).

**Results:**

At 6 months of FU indexed left ventricular end-diastolic and end-systolic volumes decreased (EDVi: 101 ± 28 mL vs. 86 ± 30 mL, *p* < 0.001; ESVi: 72 ± 23 mL vs. 55 ± 24 mL, *p* < 0.001), ejection fraction and GLS significantly improved (EF: 29 ± 6% vs. 37 ± 7%, *p* < 0.001; GLS: −9 ± 3% vs. −13 ± 4%, *p* < 0.001). A reduction of E_a_ (2.11 ± 0.91 mmHg/mL vs. 1.72 ± 0.44 mmHg/mL, *p* = 0.008) and an improvement of E_es_ (1.01 ± 0.37 mmHg/mL vs. 1.35 ± 0.6 mmHg/mL, *p* < 0.001) and VAC (2.3 ± 1.1 vs. 1.5 ± 0.7, *p* < 0.001) were observed. Re-alignment of HDFs occurred, with a reduction of diastolic L-S/A-B HDFs ratio [23 (20–35)% vs. 20 (11–28) %, *p* < 0.001].

**Conclusion:**

S/V therapy leads to a complex phenomenon of reverse remodeling involving increased myocardial contractility, HDFs distribution improvement, and afterload reduction.

## Introduction

Sacubitril/Valsartan (S/V) was proven to significantly modify the clinical course of patients with heart failure with reduced ejection fraction (HFrEF), improving symptoms, outcomes and functional capacity as a consequence of cardiac reverse remodeling (RR) (1–7). Studies on S/V induced RR focused on volumetric changes and improvement of cardiac function in terms of ejection fraction. This approach fails to describe the complex effects of S/V on cardiovascular (CV) physiopathology. Data on intraventricular pressure gradients (IVPGs) distribution are missing and those on vascular properties changes are frequently limited to hypertensive cohorts. The aim of our study was to evaluate both cardiac and vascular remodeling in a group of HFrEF patients after 6 months of therapy with S/V, in terms of volumes, contractility, IVPGs distribution, vascular properties and ventricular-arterial coupling.

## Methods

In this prospective, observational, single-center study, fifty symptomatic patients with HFrEF and an indication to receive S/V according to recommendations (8) were consecutively enrolled from January 2020 to November 2020. Before the introduction of S/V, all patients were receiving optimized medical therapy. All patients started from S/V minimal dose of 24/26 mg b.i.d. Titration up to the maximal tolerate dose was conducted every 2 weeks. Patients with diagnosis of myocarditis or who underwent coronary revascularization, cardiac resynchronization therapy (CRT) device implantation and mitral valve interventions in the last 6 months or during the follow-up period were excluded. Patients with atrial fibrillation and those who experienced death during follow-up period were excluded. All patients were in sinus rhythm and had a good acoustic window. The study was performed in accordance to the Helsinki declaration. All subjects provided written informed consent. All enrolled patients were evaluated at baseline and after 6 months of follow-up.

The study protocol included medical evaluation, blood test, transthoracic echocardiogram at baseline (before starting S/V) and after 6 months. During medical evaluation cardiovascular risk factors, past medical history, medical therapy, New York Heart Association (NYHA) functional class, systolic and diastolic blood pressure were collected. Blood test including blood count, creatinine and plasmatic potassium were collected. Trans-thoracic Echocardiography (TTE) was performed using standard equipment (*Epiq 7, Philips*). Left and right ventricle (LV and RV) dimensions, wall thickness, global and regional systolic function, indexes of diastolic function, presence and grade of valve stenosis and regurgitation were evaluated according to current guidelines (9). Three dimensional (3D) LV end-diastolic volume (EDV), LV end-systolic volume (ESV) and LV ejection fraction (EF) were calculated using an automated software *(HeartModel, Philips Healthcare)*. LV end-diastolic pressure (EDP) was also estimated (10). Comparing baseline and follow-up echocardiography, LV RR was defined as a relative increase in LVEF > 10% with a concomitant relative reduction of LVESV > 15%. Images were analyzed offline with dedicated software to assess the parameters listed below.

### Speckle Tracking Echocardiography (ST-E) Analysis

ST-E analysis was performed using an automated 2D strain analytical software *(AutoStrain, Philips Healthcare)*. The software automatically traced the endocardial border of the left ventricle in apical three, two and four chamber views, providing the mean value of endocardial global longitudinal strain (Endo-GLS).

### Non-invasive Pressure-Volume Loop Analysis (PV Loop)

PV loops were reconstructed using a dedicated software (QStrain, Medis BV, Leiden, NL). LV volumes, estimated EDP and brachial systolic and diastolic pressures were used as input. The software reconstructs the PV loop by determining the end-systolic pressure-volume relationship (ESPVR) and end-diastolic pressure-volume relationship (EDPVR) using the single-beat algorithms previously described in literature (11–13). Once the EDPVR and ESPVR are identified, the ES and ED LV volumes and systolic and diastolic brachial pressures were used to close the PV loop. Finally, the PV relation is depicted for the entire cardiac cycle where each point of the curve is described as (V_t_, P_t_). In the PV loop the classic phases of the cardiac cycle are displayed: isovolumetric contraction, ejection, isovolumetric relaxation, and diastolic filling. Based on this integrated PV loop analysis, the following hemodynamic parameters were calculated (14, 15):

- *LV systolic elastance (E*_*es*_*)*: reflecting LV contractility and representing the slope of the end-systolic pressure-volume relation (ESPVR);
- *Arterial Elastance (E*_*a*_*):* reflecting the effective arterial afterload and representing the slope of the line connecting EDV on the volume axis to the end-systolic PV point on the PV loop;
- *Ventricular-Arterial Coupling (VAC):* calculated as the ratio between E_a_/E_es_;
- S*troke Work (SW):* the external work performed by the myocardium to eject blood, computed as the area enclosed by the PV loop;
- *Mechanical Potential Energy (PE):* the energy generated within the contraction that is not converted to external work and calculated as the area enclosed by the ESPVR line, the isovolumic relaxation line and the end-diastolic pressure-volume relation (EDPVR);
- *Pressure-volume area (PVA):* the total mechanical energy generated by the contraction of the left ventricle, equal to the sum of PE and SW;
- *Work efficiency (WE):* the efficiency of the mechanical energy transfer from the ventricle to the arterial tree, expressed as the SW/PVA ratio.
- *LV end-diastolic stiffness coefficient (*β*):* representing LV end-diastolic passive filling properties and calculated as the curve-fit parameter β of the EDPVR curve.

The mathematical formulas used are reported in Supplementary Methods.

### Hemodynamic Forces (HDFs)

HDFs represent the flow forces exchanged between the blood and the endocardial boundary. HDFs were assessed using a dedicated prototype software (QStrain, Medis BV, Leiden, NL) based on a previously validated mathematical model (16). Both systolic and diastolic endocardial borders are semi-automatically traced in all the three long-axis views. LV endocardial displacement and the estimated mitral and aortic valve areas are used by the model as input data for HDF calculation. HDFs were normalized for the LV volume and expressed as a percentage of the force of gravity to compare ventricles of different sizes. HDFs were assessed over the entire cardiac cycle, in systole and diastole, and both in longitudinal (apex to base; A-B) and horizontal (latero-septal; L-S) directions. The main orientation of HDFs vector was evaluated calculating the L-S over A-B HDFs ratio (L-S/A-B HDFs ratio, %) providing a comparison between longitudinal and transverse components. HDFs directions were graphically represented using a polar histogram (17).

Based on all parameters derived by standard and advanced echocardiography the following features were described:

- *Cardiac remodeling* in terms of changes in LV volumes and systolic function, GLS, E_es_, LV diastolic stiffness, HDFs strength and distribution, non-invasive systolic pulmonary pressure, mitral regurgitation grade;
- *Vascular remodeling* in terms of changes in blood pressure measurements and E_a;_
- *Ventricular-arterial coupling and energy conversion efficiency* in terms of changes in VAC, SW, PE, PVA and WE.

### Intra-and Intra-Observer and Inter-Observer Variability

Intra-observer and inter-observer variability for HDFs and PV loop measurements were assessed in a sample of 10 patients. Two investigators measured blinded the same exam, and one investigator repeated the analysis 1 week later, blinded to the previous measurements.

### Statistical Analysis

Statistical analysis was performed with Statistical Package for Social Sciences, version 23.0 (SPSS, Chicago, IL). Variables have been analyzed to test normal distribution. They were presented as mean ± standard deviation or median and 25th−75th percentiles, when appropriate. Paired comparisons of continuous variables were performed with two-tailed paired Student's *t*-test or Wilcoxon test, when appropriate. Paired comparisons of categorical variables were conducted with the McNemar test. Interclass correlation coefficients (ICCs) were calculated to assess inter-observer and intra-observer agreement of HDFs and PV loop measurements. Differences were considered statistically significant when *p* < 0.05.

## Results

General and clinical characteristics of the whole population are depicted in Tables 1, 2. Mean age was 70 ± 12 y.o., male subjects were 41 (87%). Coronary artery disease (CAD) was the cause of HFrEF in a half of cases (26 patients, 55%). All patients were in NYHA class ≥ II (40% were in NYHA class II, 51% in NYHA class III, 9% in NYHA class IV). Before starting S/V all patients were receiving optimal medical therapy (OMT), including angiotensin converting enzyme inhibitors (ACEi) or angiotensin receptor blockers, beta blockers, diuretics and mineral-corticoid receptor antagonists. None of the patients was taking sodium-glucose cotransporter 2 inhibitors at baseline nor during follow-up period. Due to the death of 3 patients, comparisons between baseline and follow-up clinical and echocardiographic parameters were performed on a total of 47 patients. At 6 months of follow-up all patients discontinued ACEi, 33 patients (70%) were taking beta blockers, 10 (21%) mineral-corticoid receptor antagonists and 23 (49%) diuretics. One patient (2%) was re-hospitalized due to acute decompensated heart failure. The vast majority of the population experienced an improvement in symptoms as documented by a reduction of NYHA class at least of 1 point. The comparison of clinical and biochemical parameters between baseline and follow-up is showed in Table 2.

**Table 1:** General characteristics of the whole population.

**Parameters**	**HFrEF Patients *N* = 47**
**Baseline**
Age, y.o.	70 ± 12
BMI, kg/m^2^	24 ± 7
BSA, m^2^	1.9 ± 0.2
Male Sex, *n* (%)	41 (87%)
Diabetes, *n* (%)	39 (82%)
Hypertension, *n* (%)	7 (15%)
Smoke Habit, *n* (%)	24 (51%)
Dyslipidaemia, *n* (%)	28 (59%)
PMK, *n* (%)	29 (61%)
LBBB, *n* (%)	15 (31%)
CAD, *n* (%)	26 (55%)

*HFrEF, Heart Failure reduced Ejection Fraction; BMI, body mass index; BSA, body surface area; PMK, pacemaker; CAD, coronary artery disease. LBBB, left bundle branch block*.

**Table 2:** Baseline vs. follow up clinical data.

**Parameters**	**Baseline *N* = 47**	**Follow-Up *N* = 47**	***p***
SBP, mmHg	126 ± 11	119 ± 16	0.002
DBP, mmHg	78 ± 8	71 ± 8	0.001
HR, bpm	71 ± 13	67 ± 9	0.041
NYHA Class ≥ II, *n* (%)	47 (100%)	25 (53%)	<0.001
Creatinine, mg/dL	1.1 ± 0.3	1.2 ± 0.4	0.322
eGFR, ml/min	75 ± 31	73 ± 31	0.331
K+, meq/L	4.3 ± 0.5	4.4 ± 0.4	0.140

*DBP, diastolic blood pressure; eGFR, estimated glomerular filtration rate; HR, heart rate; NYHA, New York heart association; SBP, systolic blood pressure*.

### Cardiac Remodeling

**Figure 1:**
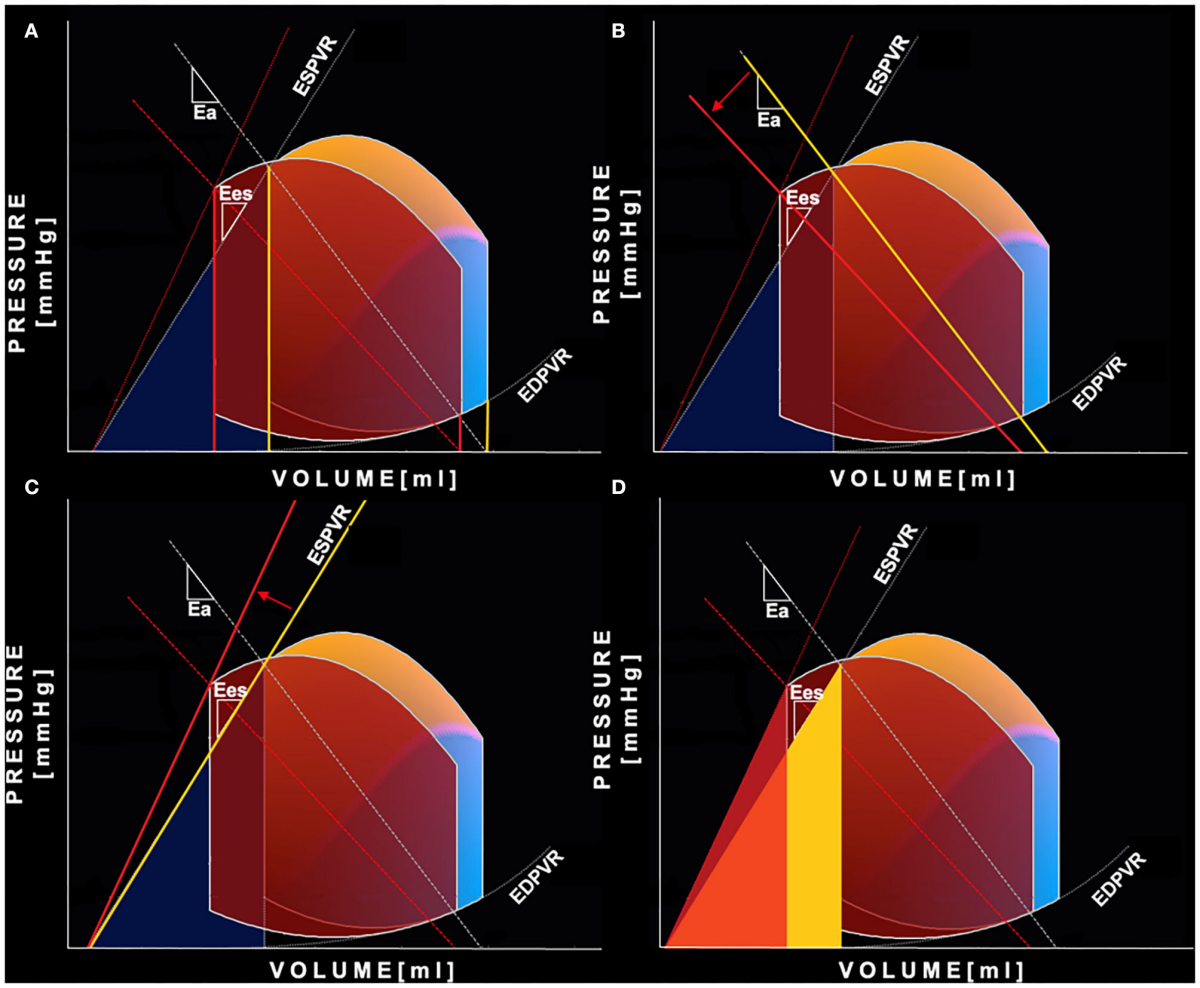
Pressure-Volume curve changes after Sacubitril-Valsartan. Representation of the non-invasive PV loop analysis of a patient before (yellow-blue PV loop) and after 6 months (red PV loop) of therapy with Sacubitril- Valsartan. **(A)** shows the reduction of both left ventricular end-systolic and end-diastolic volumes (yellow is “before,” red is “after”). The greater relative reduction of the end-systolic volume leads to an increase in stroke volume and ejection fraction. **(B)** shows decreased left ventricular afterload reflected by a less steep arterial elastance line (yellow is “before,” red is “after”); **(C)** shows increased end-systolic left ventricular elastance—steeper end-systolic pressure volume relation (yellow is “before,” red is “after”); **(D)** shows reduction of mechanical potential energy: the light yellow area is the PE before the therapy, the red is PE after the therapy. In orange is displayed the area of overlap between the aforementioned areas. PE, potential energy; PV loop, pressure-volume loop.

**Figure 2:**
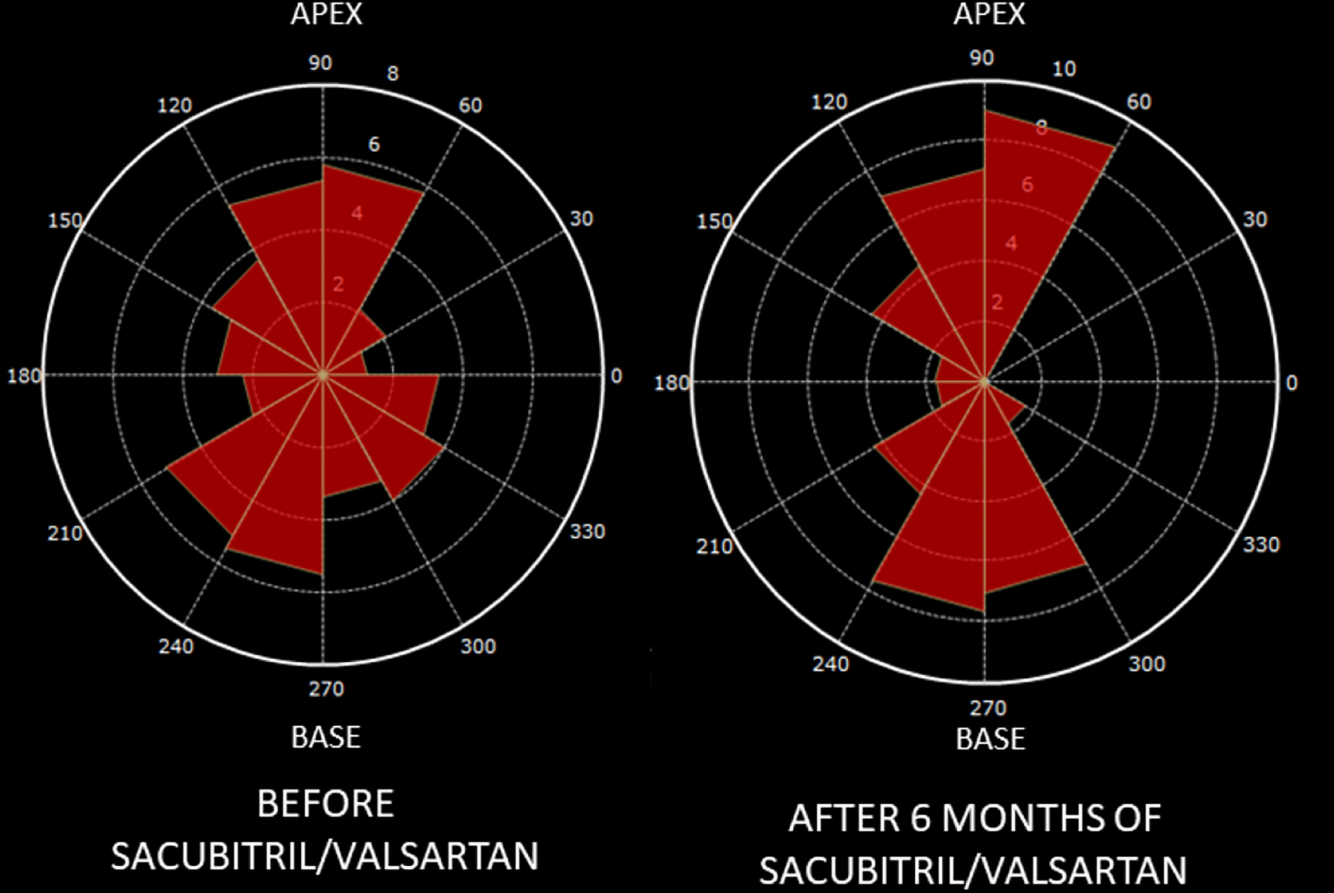
Changes in hemodynamic forces distribution after Sacubitril-Valsartan. Graphic representation on a polar histogram of left ventricular hemodynamic forces (LV-HDFs) distribution in a patient: LV-HDFs distribution assessed over the entire cardiac cycle at baseline (left) and after 6 months of therapy with sacubitril-valsartan (right), showing a re-alignment of HDFs due to an improvement of longitudinal forces (apex to base, AB) over transversal ones *(latero-septal, LS) and consequently a reduction of HDFs LS/AB ratio*. AB, apex to base; HDFs, hemodynamic forces; LV, left ventricle; LS, latero-septal; LS/AB ratio, latero-septal over apex to base ratio.

Standard and advanced echocardiographic parameters and their comparison between baseline and follow-up are depicted in Tables 3, 4. At 6 months follow-up a significant reduction in LV mass (*p* < 0.001), LVEDVi (*p* < 0.001) and LVESVi (*p* < 0.001) and an improvement in LVEF (*p* < 0.001) were observed (Table 3, Figure 1A). Twenty-two (47%) patients reached criteria of RR. At ST-E analysis, LV-GLS significantly improved. As assessed by PVloop analysis, left ventricular end-systolic elastance significantly improved (*p* < 0.001) (Table 3, Figure 1C). A significant reduction in average E/e' (*p* < 0.001) and, consequently, in EDP (*p* < 0.001) was observed. LV end-diastolic stiffness coefficient β did not change. After S/V treatment patients with moderate-to-severe mitral regurgitation had halved (p = 0.001). Left atrial volume also decreased, although its reduction was not statistically significant (*p* = 0.276). Moreover, there was a significant reduction in PASP (p =0.006). At follow-up there was also an improvement in HDFs alignment, with an increase in HDFs A-B values and consequently a significant reduction of HDFs LS/AB ratio in every phase of the cardiac cycle (Table 4; Figure 2).

**Table 3:** Baseline vs. follow-up standard echocardiography parameters, global longitudinal strain and hemodynamic parameters estimated by echocardiography.

**Parameters**	**Baseline *N* = 47**	**Follow-Up *N* = 47**	***p***
LVESVi, mL/m^2^	72 ± 23	55 ± 24	<0.001
LVEDVi, mL/m^2^	101 ± 28	86 ± 30	<0.001
LVEF, %	29 ± 6	37 ± 7	<0.001
LVMass/i, g/m^2^	191 (172–228)	172 (142–186)	<0.001
Mitral Regurgitation moderate to severe, *n* (%)	24 (51%)	11 (23%)	0.001
LAVi mL/m^2^	51 (37–61)	45 (37–58)	0.276
Average E/E'	13 (10–17)	10 (8–11)	<0.001
LVEDP, mmHg	20 ± 4	18 ± 2	<0.001
TAPSE, mm	19 ± 4	19 ± 3	0.212
Right ventricle S', cm/s	11 ± 2	11 ± 2	0.412
Tricuspid Regurgitation moderate to severe, *n* (%)	12 (25%)	7 (15%)	0.125
PASP, mmHg	36 ± 12	30 ± 6	0.006
LV-GLS-endo, %	−9 ± 3	−13 ± 4	<0.001
Ea, mmHg/mL	2.11 ± 0.91	1.72 ± 0.44	0.008
Ees, mmHg/mL	1.01 ± 0.37	1.35 ± 0.6	<0.001
VAC,-	2.3 ± 1.1	1.5 ± 0.7	<0.001
SW, Joule	0.94 ± 0.4	0.95 ± 0.31	0.899
PE, Joule	2.11 ± 0.71	1.51 ± 0.71	<0.001
PVA, Joule	3.05 ± 0.93	2.5 ± 0.87	<0.001
WE,-	0.31 ± 0.09	0.40 ± 0.10	0.001
Diastolic stiffness coefficient β, –	5,94 ± 0,47	6,10 ± 0,17	0.057

*E_a_, arterial elastance; E_es_, end-systolic ventricular elastance; EDP, end-diastolic pressure; EDVi, end diastolic volume indexed; EF, ejection fraction; ESVi, end systolic volume indexed; GLS-endo, endocardial global longitudinal strain; LAVi, left atrial volume indexed; LV, left ventricle; Mass/i, mass indexed; PASP, pulmonary arterial systolic pressure; PE, potential energy; PVA, pressure-volume area; SW, stroke work; TAPSE, tricuspid annular plane systolic excursion; VAC, ventricular-arterial coupling; WE, work efficiency*.

**Table 4:** Baseline vs. follow up echocardiographic estimated hemodynamic forces.

	**Baseline *N* = 47**	**Follow-Up *N* = 47**	***p***
**HDFs: entire cardiac cycle**			
A-B, (%)	6.1 (4.8–6.3)	8 (6.6–16)	<0.001
L-S, (%)	1.8 (1.3–2.1)	2 (1.8–2.7)	0.431
L-S/A-B HDFs Ratio, (%)	32 (30–42)	22 (6–25)	<0.001
**HDFs: systole**			
A-B, (%)	7.3 (6.8–8.2)	10.3 (7.5–24.6)	<0.001
L-S, (%)	1.7 (1.3–2.5)	2.1 (1.7–2.9)	0.013
L-S/A-B HDFs Ratio, (%)	23 (20–35)	20 (11-28)	0.001
**HDFs: diastole**			
A-B, (%)	3.6 (2.9–4.7)	6.8 (3.4–7.9)	<0.001
L-S, (%)	1.9 (1.4–2.7)	1.9 (1.2–2.7)	0.057
L-S/A-B HDFs Ratio, (%)	53 (48–72)	33 (23–38)	<0.001

*HDFs, hemodynamic forces; A-B apex to base direction; L-S, latero-septal direction; L-S/A-B HDFs Ratio, latero-septal direction over apex to base direction ratio*.

### Vascular Remodeling

Systolic and diastolic blood pressure showed a significant reduction after 6 months of therapy with S/V (*p* = 0.002 and *p* = 0.001, respectively) (Table 2). As evaluated by PV loop analysis, arterial elastance significantly reduced (*p* = 0.008) (Table 3; Figure 1B).

### Ventricular-Arterial Coupling and Energy Conversion Efficiency

After 6 months of S/V therapy, VAC significantly improved (*p* < 0.001). While SW did not differ between baseline and follow-up (*p* = 0.899), potential energy (Figure 1D) and pressure volume area significantly reduced (*p* < 0.001 and *p* < 0.001, respectively). Thus, WE significantly improved (*p* = 0.001).

### Intra- and Inter-Observer Agreement

Both intra- and inter-observer agreement were good to excellent for all the parameters. ICCs are reported in Supplementary Table 1.

## Discussion

In this study, the occurrence of both cardiac and vascular remodeling in 47 HFrEF patients after 6 months of therapy with S/V was described. The strength of our study is the comprehensive evaluation of CV remodeling after S/V not only in terms of volumetric remodeling but also, for the first time, in terms of deformation, intraventricular pressure gradients, hemodynamics and vascular remodeling using non-invasive methods for PV loop analysis and intraventricular HDFs assessment. Conventionally, cardiovascular remodeling has been identified with reverse LV remodeling in terms of “volumetric” parameters: reduction of LVEDV, LVESV and improvement of LVEF (6, 18, 19). Recently, S/V has been demonstrated to induce improvement in LV function in terms of muscular deformation by ST-E (18). However, the description of changes in the cardiovascular mechanics via ejection fraction and volumes is a crude simplification. This approach fails to describe the complexity of a muscular pump interacting with the intracavitary blood flow and coupled with the arterial tree.

### Cardiac Remodeling

In the overall cohort, we observed a significant improvement in LVEF and an important reduction in LVESVi and LVEDVi. A concomitant end-systolic volumes reduction and EF improvement (LV RR) was observed in 47% of the total cohort. Our results are in line with previous reports in literature. Several studies have shown that the therapy with S/V induces cardiac remodeling (3, 4) with a dose-dependent effect (3). Compared with ACEi, angiotensin receptor neprilisyn inhibitors (ARNIs) were found to induce more frequently RR in terms of EF improvement and volumes reduction, clinically reflected by a lower NYHA class and better performances at 6-minutes walking test (6MWT) (5). In our study, the LVEF improvement is supported by the higher systolic myocardial deformation and increased intrinsic contractility observed after 6 months (significant increase in both GLS and E_es_). These findings are in line with previous reports investigating the effect of S/V on myocardial strain (18, 20). Preclinical evidences showed that Sacubitril and Valsartan have a synergic effect, attenuating cardiomyocyte cell death, hypertrophy and impaired contractility (21). Moreover, the natriuretic and diuretic effects of S/V could reduce cardiac preload, allowing the heart to work on the most efficient part of the Frank-Starling curve and improving the stroke volume. Changes in cardiac preload, together with LV volume reduction, influence also diastolic properties. Precisely, we documented a reduction in LV EDP without a significant reduction in LV stiffness coefficient (β). This may be counterintuitive if we do not focus on the diastolic properties of the failing heart. In patients with HFrEF, the LV is characterized by high diastolic capacitance and stiffness. The end diastolic point of the PV loop is determined not only by the curve fit parameters of the EDPVR but it is also extremely influenced by the volume status. Even in the absence of changes in stiffness, a preload reduction causes a volume-dependent decrease in EDP and the end-diastolic point (V_ED_,P_ED_) shifts down-left along the EDPVR. Thus, the decrease in filling pressure after S/V are mainly due to a reduction in volume overload. We should acknowledge that the follow-up period in our study is relatively short and possible effects of S/V on LV diastolic stiffness may need a longer time. Another known beneficial effect of S/V is the reduction in severity of functional mitral regurgitation due to lower cardiac preload, reduced LV volumes and increased LV systolic function leading to a rebalancing of closing and tethering forces. All the aforementioned beneficial effects, summed with a possible role of S/V in reducing the pulmonary vascular tone, concur to an improvement of post-capillary pulmonary pressures (22, 23).

In our report, we documented a realignment of HDFs during S/V therapy. HDFs are forces exchanged between the blood and the myocardium during the cardiac cycle. Blood flows into the cardiac chambers because of intra-ventricular pressure gradients (IVPGs), changing throughout the entire cardiac cycle. They are generated by the totality of the cardiac structure (opening and closing valves, contracting and relaxing myocardium, vessels) (24). Recently, non-invasive HDFs analysis, using the application of a mathematical model to echocardiographic or magnetic resonance “cine” images, has been validated (16, 25). HDFs magnitude and alignment have been recently proposed as novel markers of cardiac function. Briefly, in normal hearts HDFs are mainly directed in apex-to-base (or longitudinal) direction, while latero-septal (or transversal) HDFs are significantly weaker. Misalignment of HDFs has been reported in abnormal cardiac conditions and related to dyssynchrony and regional heterogeneity in myocardial contraction and relaxation (26–28). In HFrEF patients HDFs are significantly lower and misaligned, diverging from the normal apex-to-base direction toward the latero-septal one (29). A recent pathophysiological model suggested a link between HDFs misalignment/re-alignment and adverse-remodeling/reverse-remodeling (27, 28). Cardiac endothelial mechano-receptors can distinguish changes in shear stress vectors (tangential vs. perpendicular direction) and activate ultrastructural adaptive responses, such as turnover of contractile proteins and regulation of myofibril orientation (30).

### Vascular Remodeling

After 6 months of therapy with S/V, both SBP and DBP significantly improved. Moreover, P/V loop analysis showed a significant reduction in E_a_. Reduction of blood pressure may be explained not only by the diuretic and natriuretic effect but also by vascular remodeling. S/V was proven to improve endothelium-dependent and independent vasorelaxation (31) and to reduce arterial stiffness (32). Thus, S/V could significantly change both the static (total vascular resistance) and pulsatile components (e.g., total wave reflections) of the total arterial load (33). These biological effects are also mediated by an increased availability of natriuretic peptides with vasoactive properties and by modulation of both sympathetic nervous system and renin-angiotensin system (34, 35).

### Ventricular-Arterial Coupling and Energy Conversion Efficiency

The ultimate effect of S/V is an amelioration of ventricular-arterial coupling, as demonstrated in our cohort of patients by a significant reduction in E_a_/E_es_ ratio. Arterial load and stiffness are closely linked with systolic and diastolic function and LV hypertrophy (14). Improvements in arterial compliance, peripheral resistance, and wave reflections optimize LV afterload. This reduces early and peak systolic myocardial stress and oxygen demand. Complementary improvement in LV systolic function and organ perfusion lead to neuro-humoral and sympathetic modulation, contrasting vascular dysfunction (36). The amelioration of E_a_, E_es_, and VAC is the physiopathological cause of the improvement of LVEF, used as a surrogate of systolic function. Actually, the relationship between VAC and EF can be mathematically described as LVEF = E_es_/(E_es_+Ea), showing how EF is affected both by inotropic state and total afterload (37). The optimization of VAC improves the energetic efficiency of the cardiovascular system. In order to maintain adequate stroke volume and peripheral perfusion, the failing heart uses compensation mechanisms, which increase energy consumption. However, a significant part of the energetic expenditure is wasted and do not concur to blood ejection (38). During S/V therapy, we observed a reduction in PE and PVA while SW did not differ. PVA reduction reflects a decrease in the total mechanical energy of contraction and consequently in myocardial oxygen consumption, while PE is the amount of energy not converted to external work (or ejection, equivalently) (39, 40). Consequently, during S/V therapy WE improves, reflecting a more efficient mechanical energy transfer from the ventricle to the arterial system. It has been demonstrated that WE is a monotonic function of VAC and can be formulated as SW/PVA = 1/[1+(E_a_/E_es_)/2] (41). An optimization of contractile state and/or a reduction of arterial afterload improves VAC, reduces energetic demand and increases WE.

### Limitations

The most important limit of our study is the small sample size. Therefore, our results, especially those regarding HDFs estimation, should be considered as preliminary observations. Moreover, HDFs computation by echocardiography depends on image quality and it has to be considered as an estimation of real hemodynamic forces. Vascular afterload was assessed only with PV loop derived parameters. Finally, significant clinical outcomes were not evaluated, due to the limited number of major cardiac events observed during follow-up.

## Conclusions

After 6 months of S/V therapy, cardiovascular remodeling was observed in our cohort of HFrEF patients, in terms of volumes reduction, increased myocardial contractility, intraventricular-pressure gradients distribution improvement and optimization of vascular afterload. The ultimate effect is an amelioration of ventricular-arterial coupling and mechanical energy conversion efficiency. Our findings highlight the pleiotropic effect of S/V therapy generating a virtuous circle of both cardiac and vascular remodeling.

## Data Availability Statement

The raw data supporting the conclusions of this article will be made available by the authors, without undue reservation.

## Ethics Statement

The studies involving human participants were reviewed and approved by Policlinico Umberto I. The patients/participants provided their written informed consent to participate in this study.

## Author Contributions

SM, DF, LA, and VM contributed to conception and design of the study. MS and FL organized the database. SM performed the statistical analysis and wrote the first draft of the manuscript. DF, SC, MN, MM, LIB, GB, GT, GP, and CDV wrote sections of the manuscript. All authors contributed to manuscript revision, read, and approved the submitted version.

## Funding

This study received funding from Novartis. The funder was not involved in the study design, collection, analysis, interpretation of data, the writing of this article or the decision to submit it for publication.

## Conflict of Interest

The authors declare that the research was conducted in the absence of any commercial or financial relationships that could be construed as a potential conflict of interest.

## Publisher's Note

All claims expressed in this article are solely those of the authors and do not necessarily represent those of their affiliated organizations, or those of the publisher, the editors and the reviewers. Any product that may be evaluated in this article, or claim that may be made by its manufacturer, is not guaranteed or endorsed by the publisher.
