# Supplementary material for: Cardiac and Vascular Remodeling After 6 Months of Therapy With Sacubitril/Valsartan: Mechanistic Insights From Advanced Echocardiographic Analysis
Source: Front Cardiovasc Med. 2022 May 18;9:883769. doi: 10.3389/fcvm.2022.883769 (PMC9157573; doi:10.3389/fcvm.2022.883769)
Supplement: Supplementary file 1 [file Data_Sheet_1.docx]

Supplementary Material

**1.Supplementary Data**

The following data has been provided as supporting material due to limited space in the main manuscript.

Although not essential for the understanding of the paper, the authors felt that this will complement the main manuscript.

**Non-invasive P-V loop analysis: mathematical formulas.**

The pressure (P)- volume (V) loop was reconstructed starting from the end-systolic pressure-volume relationship (ESPVR) and the end-diastolic pressure-volume relationship (EDPVR).

The ESPVR is constructed as

$P=E_{es}\left( V-V_{0} \right)$

where $E_{es}$ is the ventricular elastance.

The reference volume $V_{0}=V_{ES}-\frac{P_{ES}}{E_{es}}$ is just the intercept with the zero-pressure axis.

According to the single-beat algorithm by Chen et al. E_es_ is calculated as

$E_{es}=\frac{1}{SV}\left( \frac{P_{D}}{E_{Nd}}-P_{ES} \right)$

with the constraint that this value is bounded by the limit $E_{es}>\frac{P_{ES}}{V_{ES}}$ to ensure that $V_{0}>0$. In this equation P_D_ and P_ES_ are the diastolic and end-systolic blood pressures, E_Nd_ is the estimated normalized ventricular elastance at the onset of ejection, SV is stroke volume.

P_ES_ is estimated as 90% of the brachial systolic pressure ($P_{S}$).

E_Nd_ is described by the following formula:

$E_{Nd}=0.0275-0.165EF+0.3656\frac{P_{D}}{P_{ES}}+0.515\sum_{0}^{7} a_{n}\left( 1+\frac{t_{IVC}}{t_{ejection}} \right)^{n}$ .

where EF is the ejection fraction, t_IVC_ ad T_ejection_ are the iso-volumic contraction (IVC) time and the ejection time, respectively. The last corrective term is made with the following coefficients

$$\boldsymbol{a}=\left[ \begin{aligned} +0.35695 \\ -7.2266 \\ +74.249 \\ -307.39 \\ +684.54 \\ -856.92 \\ +571.95 \\ -159.10 \end{aligned} \right]$$

The ratio between IVC time and ejection time is measured by aortic Doppler or can be computed directly from the speckle tracking information using mass conservation and mitral size.

Once the ESPVR is derived, the EDPVR is described using the nonlinear expression

$P=\alpha V^{\beta}$

using the previously described step-by-step algorithm by Klotz et al.

First of all the end-diastolic pressure is estimated by

$P_{ED}=11.96+0.596\frac{E}{e^{'}}$,

where E/e’ is the ratio between early mitral inflow velocity and mitral annular early diastolic velocity.

Secondly, the partial volumes are computed

$V_{0}=\left( 0.6-0.006P_{ED} \right)V_{ED}$, $\begin{matrix} V_{30}=V_{0}\left( V_{ED}-V_{0} \right)\left( \frac{A}{P_{ED}} \right)^{\frac{1}{b}} & whereA=27.78\pm0.3[mmHg]andb=2.76\pm0.05[-] \end{matrix}$

Finally, the two parameters $\alpha$ and $\beta$ are evaluated as follows.

If $P_{ED}\geq22$,

$\begin{matrix} \beta=\frac{log\frac{P_{ED}}{15}}{log\frac{V_{ED}}{V_{15}}}, & \alpha=P_{ED}{V_{ED}}^{-\beta}; & withV_{15}=V_{0}+0.8\left( V_{30}-V_{0} \right) \end{matrix}$,

Otherwise, when $P_{ED}<22$

$\begin{matrix} \beta=\frac{log\frac{P_{ED}}{30}}{log\frac{V_{ED}}{V_{30}}}, & \alpha=30{V_{30}}^{-\beta} \end{matrix}$.

Once the ED and ES PV relationships are identified, a pressure-volume relationship (PV Loop) can be depicted for the entire cycle. The PV loop is made by four branches in the pressure-volume plane, (i) the systolic contraction is a curve that ranges from the point $\left( V_{ED},P_{ES} \right)$ to the point $\left( V_{ES},P_{ES} \right)$ with a peak pressure in between equal to $P_{S}$; (ii) the isovolumic relaxation assumed to be a vertical line from $\left( V_{ES},P_{ES} \right)$ to $\left( V_{ES},P_{ED} \right)$; (iii) the diastolic filling is a curve that starts from $\left( V_{ES},P_{ED} \right)$ and approaches tangentially the EDPVR when it reaches the point $\left( V_{ED},P_{ED} \right)$; (iv) the isovolumic contraction assumed a vertical line from $\left( V_{ED},P_{ED} \right)$ to $\left( V_{ED},P_{ES} \right)$.

The main properties of the PV loop are consequently derived. In addition to the ventricular elastance, the arterial elastance $E_{a}$ is evaluated as the ratio between end-systolic pressure and stroke volume,

$E_{a}=\frac{P_{ES}}{V_{ED}-V_{ES}}$ .

From the ratio between the two elastance values the ventricular-arterial coupling is computed as

$VAC=\frac{E_{a}}{E_{es}}$.

The energetic properties are summarized in term stroke work ($SW$) given by the area of the PV loop and the potential energy ($PE$) given by the area of the triangle made by the ESPVR and the PV loop The PE is expressed as $PE=\frac{1}{2}P_{ED}\left( V_{ES}-V_{0} \right)$.

The total PV area (PVA) is calculated as the sum of SW and PE

$PVA=SW+PE$,

and the work efficiency (WE) by dividing the stroke work for the PV area

$WE=\frac{SW}{PVA}$.

**2.Supplementary Table**

**Table 1. Intra‐ and inter‐observer agreement for hemodynamic forces and PV-loop measurements.**

| **Parameters** | **Inter‐observer**  **agreement** | **P** | **Intra‐observer**  **agreement** | **P** |
| --- | --- | --- | --- | --- |
| **Hemodynamic forces: entire heart cycle** | | | | |
| **A-B, (%)** | 0.85 (0.36-0.96) | 0.006 | 0.82 (0.22-0.96) | 0.014 |
| **L-S, (%)** | 0.87 (0.14-0.97) | 0.001 | 0.76 (0.015-0.94) | 0.030 |
| **L-S/A-B HDF Ratio, (%)** | 0.76 (0.06-0.94) | 0.018 | 0.79 (0.062-0.95) | 0.024 |
| **PV-loop** | | | | |
| **Ea, mmHg/mL** | 0.76 (0.19-0.95) | 0.004 | 0.95 (0.78-0.98) | 0.001 |
| **Ees, mmHg/mL** | 0.75 (0.21-0.92) | 0.031 | 0.78 (0.1-0.95) | 0.032 |
| **PVA, Joule** | 0.86 (0.03-0.97) | 0.001 | 0.97 (0.86-0.99) | 0.001 |
| **SW, Joule** | 0.85 (0.04-0.97) | 0.001 | 0.98 (0.91-0.99) | 0.001 |
| **PE, Joule** | 0.76 (0.08-0.94) | 0.018 | 0.9 (0.53-0.97) | 0.003 |

*HDFs: hemodynamic forces; A-B apex to base direction; L-S: latero-septal direction; L-S/A-B HDFs Ratio: latero-septal direction over apex to base direction ratio; E_a_: arterial elastance; E_es_: end-systolic ventricular elastance; PE: potential energy; PVA: pressure-volume area; SW: stroke work.*
